# A Novel Approach for Evaluating the Contraction of Hypo-Peritectic Steels during Initial Solidification by Surface Roughness

**DOI:** 10.3390/ma11040571

**Published:** 2018-04-07

**Authors:** Junli Guo, Guanghua Wen, Dazhi Pu, Ping Tang

**Affiliations:** College of Materials Science and Engineering, Chongqing University, Chongqing 400044, China; wengh@cqu.edu.cn (G.W.); 20160902065@cqu.edu.cn (D.P.); tping@cqu.edu.cn (P.T.)

**Keywords:** hypo-peritectic steels, phase transformation, contraction, surface roughness, continuous casting

## Abstract

The contraction of peritectic steels in the initial solidification has an important influence on the formation of surface defects of continuously cast slabs. In order to understand the contraction behavior of the initial solidification of steels in the mold, the solidification process and surface roughness in a commercial hypo-peritectic and several non-peritectic steels were investigated using Confocal Scanning Laser Microscope (CSLM). The massive transformation of delta-Fe (δ) to austenite (γ) was documented in the hypo-peritectic steel, which caused surface wrinkles and greatly increases the surface roughness of samples in the experiments. Surface roughness (R_a(δ→γ)_) was calculated to evaluate the contraction level of the hypo-peritectic steel due to δ–γ transformation. The result shows that the surface roughness method can facilitate the estimation of the contraction level of peritectic transformation over a wide range of cooling rates.

## 1. Introduction

It is well known that hypo-peritectic steels are more sensitive to deep oscillations, surface shape variations, cracks, and even breakouts than low or high-carbon counterparts during continuous casting, especially at high casting speeds [1,2,3,4,5]. The surface quality problems are strongly related to the complicated peritectic transition process, which leads to nonuniform volume contraction of hypo-peritectic steels during the initial solidifying stage in the mold [4,5,6,7,8]. However, because peritectic steels have excellent mechanical properties, such as Advanced High Strength Steel and High Strength Low Alloy [9,10], and low manufacturing costs, they are still produced preferentially, even though surface defects are often encountered. Currently, there is great uncertainty about the kinetic properties of the peritectic phase transition [11], therefore, reduced speed and reasonable selection of mold flux are currently the major options to minimize defects 4. In order to increase the productivity and improve the surface quality, it is crucial to understand the shrinkage behavior of the peritectic phase transition during the initial solidification of peritectic steels.

The peritectic solidification process has been defined as two separate components by Kerr et al. [12]: In the Fe–C system at 1768 K, the austenite (γ) nucleates and grows along the liquid (L)–ferrite (δ) interface, namely L+δ→γ, until δ and L are completely separated by the advancing γ, which is the peritectic reaction. The subsequent growth of γ into the δ and L phases, respectively, is classed as the peritectic transformations. Generally, severe volume shrinkage and defect susceptibility occur when casting steels with carbon content between 0.10%C and 0.18%C, because the peritectic transformation of δ to γ coincides with the final solidification and ends in the solid during the early solidification stage, which can enhance the thermal contraction [7,8,9,13]. Therefore, the degree of contraction and defect susceptibility of steels are always evaluated according to the relationship between the carbon content and the position of the peritectic point. Steels are made of a multicomponent material alloying elements and the peritectic reaction regions are significantly influenced by the alloy elements. Several methods are used to characterize steel grade. One is the calculation of carbon equivalent, which is the most frequently used method; another is the use of thermodynamic calculation software, such as FactSage [14] and ThermoCalc [15], which predict the phase transformation of steels under equilibrium conditions. For some new steels, Differential Scanning Calorimetric (DSC) is also used to determine the solidification characteristics because software calculation accuracy is limited by the extent of the database used [16,17].

All of the methods mentioned above are mainly based on thermodynamic analysis under equilibrium conditions, however, the practical continuous casting process is far from equilibrium conditions. The peritectic phase transition of steels is affected by rapid cooling, and an increase in cooling rate promotes defect sensitivity since it promotes a quick structural evolution [18]. Shibata et al. [19] first reported in-situ observation experiments of the peritectic steels by using the high-temperature CSLM technique. The results showed that the rate of peritectic reaction and phase transformation increase with increased cooling rate. After that, a concentric solidification technique was developed [20], many researchers [9,21,22,23] investigated the change of cooling rate and carbon content on the solidifying of peritectic steels, and they found that there are three models of peritectic transformation; the progression of a planar interface, a cellular/dendritic transformation, and a massive transformation. These models represent different contraction rates. Griesser et al. [23] pointed out that differences in the modes of peritectic transformation and contraction rate (the progression velocity of interface) in a local area lead to uneven shell shrinkage. However, when the progression velocity of the δ–γ interface is used to further study the phase transition contraction, it is always difficult to quantity the interface movement at higher cooling rates because the δ–γ interface velocity is too fast to be clearly observed even at the cooling rate of 20 K/min for hypo-peritectic steels [22], which is significantly smaller than the maximum cooling rate in the meniscus region of the continuous casting mold up to 3600 K/min [24]. Moreover, the significant fluctuation of the local velocity is also problematic [11].

In addition, some valid numerical works [25,26,27,28,29,30] which combined multiple factors, such as shrinkage of the interfacial gap and the thermomechanical behavior of the solidifying shell, have also described the solidification in the mold. Park et al. [25] revealed the strain and stress distribution of the shell under different conditions by establishing a three-dimensional steady-state thermal-mechanical coupling model. Li and Thomas et al. [26] coupled the CON1D model [27] with the elastic-viscoplastic constitutive models to simulate temperature, stress, and shape deformation of the shell during the continuous casting process. For the solidification of hypo-peritectic steels, δ–γ transformation occurs instantly because of massive transformation, rather than diffusional growth of γ acting on the semi-solidified shell. Therefore, it is not easy to accurately determine the shrinkage that is important for the heat transfer variation. Measurement of the stress and strain during solidification is a direct way to analyze the relationship between phase transformation shrinkage and the cooling conditions, for example, by use of high temperature tensile test apparatus to measure the strength during solidification [31] and submerged split-chill tensile (SSCT) testing for measurement of tensile forces [32]. However, the former needs to accurately control the direction of dendrite growth with near equilibrium conditions of low cooling rate, whereas ensuring the uniform shell growth around the mold is required for SSCT tests. Thus, discrete and imprecise data are easily obtained owing to the difficulty of controlling the process. Therefore, it is necessary to find a method which can accurately evaluate the effect of cooling rate on peritectic transformation contraction to enhance our understanding of the shrinkage behavior of peritectic steels under practical conditions.

Peritectic transformation causes uneven deformation of the shell during the initial solidification, meanwhile, wrinkles are also generated on the solidified surface on the microscopic scale [21,22,23]. These wrinkles are caused by contraction strain and the relationship between stress strain and wrinkles (surface roughness) has been studied in several materials, including steels [33], brass sheets [34], and aluminum alloys [35]. These results all show that small scale strain localization near the material surface causes a linearly-related change in the surface roughness. In this study, based on the characteristics of the solidification surface wrinkles, the surface roughness of several steels are discussed in combination with the solidification process. The surface roughness method is put forward to provide a better insight into the shrinkage of hypo-peritectic steels during the initial solidification stage.

## 2. Materials and Methods

### 2.1. Materials

In order to illustrate the potential of the surface roughness method, a commercial hypo-peritectic steel and several non-peritectic steel samples were selected to investigate and discuss. The chemical compositions of the steel samples were analyzed by optical emission spectrometry (Table 1).

### 2.2. Confocal Scanning Laser Microscope (CSLM)

A Yonekura VL2000DX-SVF17SP CSLM [36] was used to heat and cool the steel. The sample in the alumina crucible, which is placed on a Pt-sample holder located at the focal point of the IR beam, is heated by the IR radiation. The R type thermocouple installed in the Pt-sample holders is used to measure and control the temperature. The sample can be heated to 1600 °C in less than 30 s with the highest heating rate and the highest cooling rate can approach −100 °C/s by a rapid cooling mechanism using high pressure He gas when necessary. High-temperature CSLM has been described elsewhere by several researches [19,20,21]. A standard objective lens is used to measure surface roughness (R_a_) with the computer software (LMeye) (Yonekura, Yokohama, Kanagawa, Japan) to get the 3D image information of samples by CSLM. The measurement principle [37] is as follow: the light source, the sample, and the detector are placed at the conjugate points; the laser beam from the light source reaches the sample through the spectroscope technology. Then, the light reflected to the beam-splitter is refracted to a detector through the detection pinhole. Because the detection pinhole only allows the light reflected from the focal plane on the sample to pass, the object can be divided into a number of layer-by-layer scans, which yields a 3D measurement.

**Figure 1:**
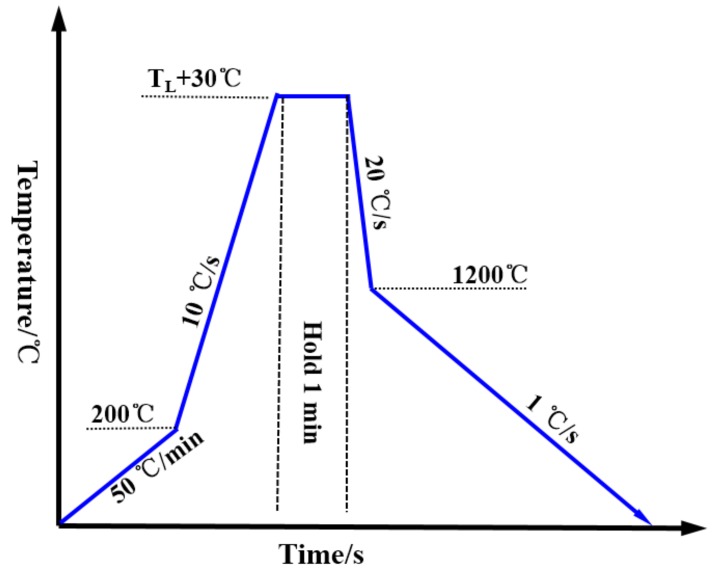
Thermal scheme.

In the study, the sample size was a 7.8-mm-diameter cylinder with a height of 2.5 mm. Figure 1 is the thermal scheme. The sample was heated to T_L_+30 °C at first and subsequently held for 1 min, after which a quick cooling rate of −20 °C/s was applied to cool the samples to 1200 °C. Finally, the sample was cooled to room temperature with a cooling rate of 1 °C/s. The cooling air was high purity argon (6N) and a video was recorded at 20 frames per second during cooling. For the surface roughness measurement, the 3D surface morphology of samples were scanned, firstly by CSLM with the standard objective lens (×1000 magnification), then we manually choose the measuring region by “scoring a line” (the method for selecting the measurement line is described in Section 3.3.1) and the surface outline and surface roughness can be obtained automatically through the software. To ensure date reliability and result reproducibility, at least 15 different locations in 3 fields of each sample were measured to calculate the surface roughness.

## 3. Result and Discussion

### 3.1. Solidification Characteristics

**Figure 2:**
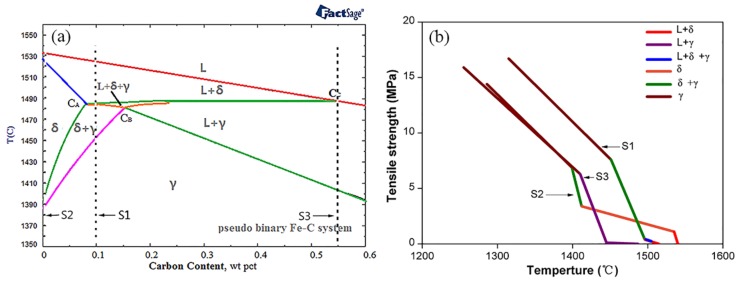
FactSage calculation of pseudo-binary Fe–C diagram (**a**) and the relationship between temperature and tensile strength during solidification (**b**).

The pseudo-binary Fe–C diagram was determined by using FactSage 6.2 (Figure 2a). The solidification sequence of the hypo-peritectic steel (S1) is significantly different from other steels during the initial solidification stage, where the transformation of δ to γ occurs at the final solidification and ends in the solid. As the phase transformation starts to occur, the solidifying shell is still in the mushy zone and begins to withstand deformation and transfers pressure at the macroscopic level, whereas there are still some liquid droplets remaining in the dendrite arms at the microstructure level, which greatly reduces the strength of the solidifying metal. Figure 2b shows the predicted variation of tensile strength with temperature under equilibrium conditions for several steels, and the calculation process referred to in the literature 18. The tensile strength of the hypo-peritectic steel is almost zero when the δ–γ transformation occurs, so the shell in the meniscus region deforms easily due to shrinkage stress. By contrast, although ultra-low carbon steel also exhibits a δ–γ transformation, because the solidification has finished and the shell exhibits a certain strength (σ = 4 MPa), contraction deformation is greatly reduced greatly. For high-carbon steel, a different process of γ nucleation occurs and its strength (σ = 6 MPa) is greater than that of hypo-peritectic and ultra-low carbon steels after solidification, which results in a harder surface.

### 3.2. Initial Solidification Process of Steels during Cooling

Here, the solidification processes of three kinds of steels were observed by CSLM, and the solidification processes were analyzed in combination with the evolution of the microstructure.

#### 3.2.1. Hypo-Peritectic Steel

**Figure 3:**
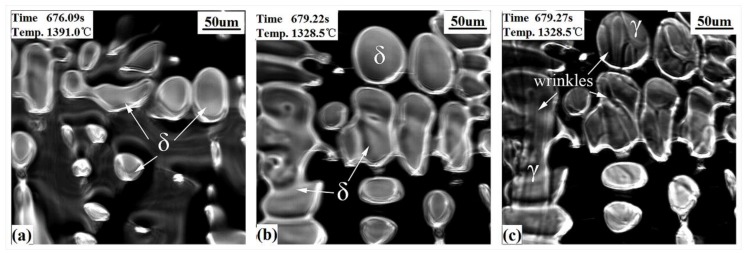
CSLM results of the hypo-peritectic steel (S1) at cooling rate of 20 °C/s. (**a**) δ growth; (**b**,**c**) before and after δ–γ transformation.

CSLM images (Figure 3) show the events which occur upon the surface of the hypo-peritectic steel during cooling. The undercooling was achieved with an initial cooling rate of −20 °C/s , which delayed the nucleation and transformation. Table 2 shows the comparison of the temperature of nucleation and the δ–γ transformation between theoretical and experimental cooling. δ began to nucleate from the liquid phase at 1488.3 °C and then constantly increased. At the same time, the liquid contraction caused the δ grains to rise and hollows to appear on the steel surface (the black region in Figure 3a,b). The solidification process in hollows cannot be observed because the reflected light from the hollows cannot be reflected back. According to the solidification sequence, after the peritectic reaction has finished, the δ dendrite core begin to transform to single γ at lower temperatures with because the large cooling rate creates a thermodynamic driving force to undergo massive transformation rather than diffusional growth 23. Figure 3b,c exhibits the contrast of the transformation before and after, where surface wrinkles are generated on crystals at 1328.5 °C within 0.05 s (two frames on video tape). Judging descriptions in the literature [19,21,22,23] and the solidus line in the phase diagram, the wrinkles are believed to illustrate the fact that the peritectic δ–γ transformation has finished as a result of the non-uniform transformation contraction. The definite process was impossible to determine because it occurs too quickly.

**Figure 4:**
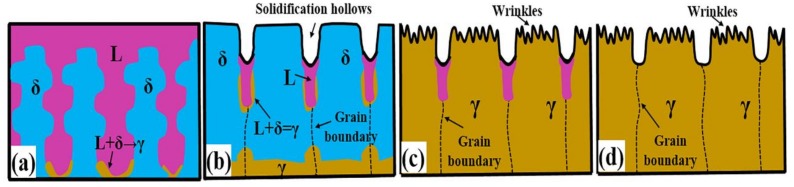
Schematic diagram of the surface solidifying process for hypo-peritectic steels. (**a**) Peritectic reaction; (**b**,**c**) Before and after δ–γ transformation; (**d**) Solidification finished.

In the process of phase transformation, one thing to note is that the previously solidified surface profile will not be completely subverted by the peritectic transformation contraction because its shrinkage is much smaller than the solidification shrinkage. In addition, the corrosion of the longitudinal section shows that primary dendrites grow in a certain direction and the dendrite spacing is consistent with the rise in grain spacing on the surface, consistent with the literature [38]. Figure 4 is a schematic diagram describing the processes of surface solidification and microstructure evolution for hypo-peritectic steels (S1). There are convexities formed representing the priority delta-Fe during solidification and the initial grain boundaries are located in hollows, and then the peritectic transformation result in the contraction wrinkles emerging on crystals.

#### 3.2.2. Ultra-Low Carbon Steel

**Figure 5:**
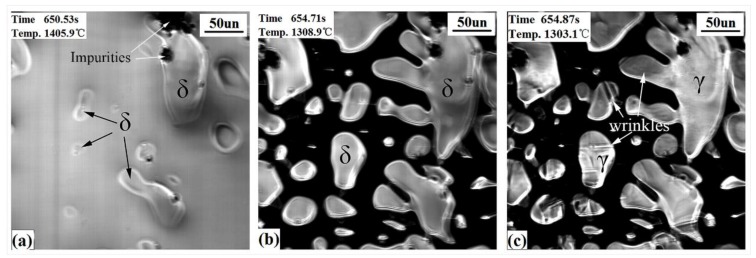
CSLM results of the ultra-low carbon steel (S2) at cooling rate of 20 °C/s. (**a**) δ growth; (**b**,**c**) before and after δ–γ transformation.

Figure 5 shows the in-situ observational results for ultra-low carbon steel (S2). At first, δ began to precipitate at 1462.5 °C and then grow gradually (Figure 5a), following a region of single δ. There are also some forming δ crystals and hollows being generated during the liquid solidifying contraction. The transition of δ to γ occurs at 1308.9 °C, which took about 0.16 s (Figure 5b,c). Surface wrinkles and contraction lines were produced on the crystals because of the δ-γ transformation (Figure 5c). As we observed for the ultra-low carbon steel, surface deformation is relatively smaller than for the hypo-peritectic steel, from comparing the shallow shrinkage lines of S2 with the shrinkage grooves of S1. This is due to the higher tensile strength of the shell and the lower the temperature at which the phase transition occurs.

#### 3.2.3. High Carbon Steel

**Figure 6:**
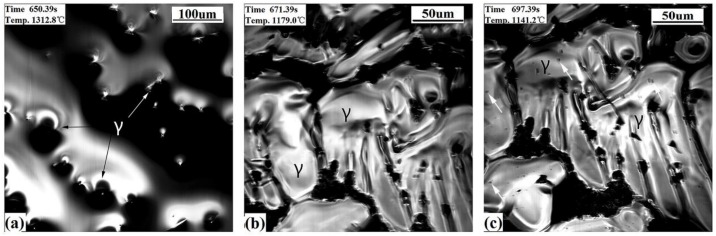
CSLM results of the high carbon steel (S3) at a cooling rate of 20 °C/s. (**a**) γ growth; (**b**) γ phases; (**c**) precipitates emerging.

Figure 6 shows the CSLM results of the high carbon steel. The γ dendrites directly appear from the liquid at 1338.5 °C and are followed by γ growing (Figure 6a) until the steel solidifies completely. There were no wrinkles found on the sample’s surface, and precipitates (see arrow) appeared from the matrix at 1141.2 °C (Figure 6c), which is due to the lack of a solid phase transition for this steel during the initial stage of solidification.

### 3.3. Measurement of Surface Roughness

#### 3.3.1. Measurement Regions

The volume shrinkage due to the δ–γ transformation is believed to be the key cause of surface defects in peritectic steels (S3). According to Section 3.2.1, the deeper hollows that appeared were caused by the solidification contraction and are independent of the δ–γ transformation. Therefore, in order to evaluate the volume shrinkage degree caused by phase transition, measurement of the variation in surface roughness on the grains is a valid strategy to avoid the influence of the liquid and the solidification contraction.

**Figure 7:**
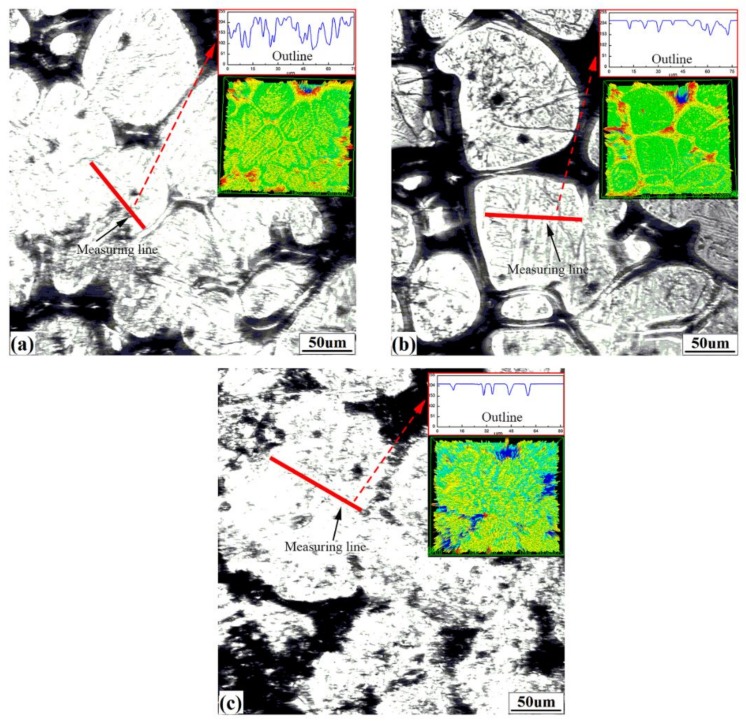
Surface morphology obtained by CSLM: (**a**) S1, (**b**) S2, (**c**) S3. The insets are corresponding magnified outlines and 3D confocal images.

Figure 7 shows the surface morphologies and 3D images obtained by CSLM for the three steels. The microstructure surfaces of steels are composed of solidifying hollows and raised crystals. After completing 3D scanning, surface outlines and surface roughness can be extracted by manually “drawing lines” on the crystals’ surfaces, where measuring lines should exist on “one grain” and not extend into hollows as far as possible, as shown in Figure 7a–c by red solid lines. The outlines are displayed in the upper right of Figure 7a–c. There are significantly more fluctuations in the hypo-peritectic steel than in other steels. This variation is closely related to their solidification characteristics.

#### 3.3.2. Surface Roughness

**Figure 8:**
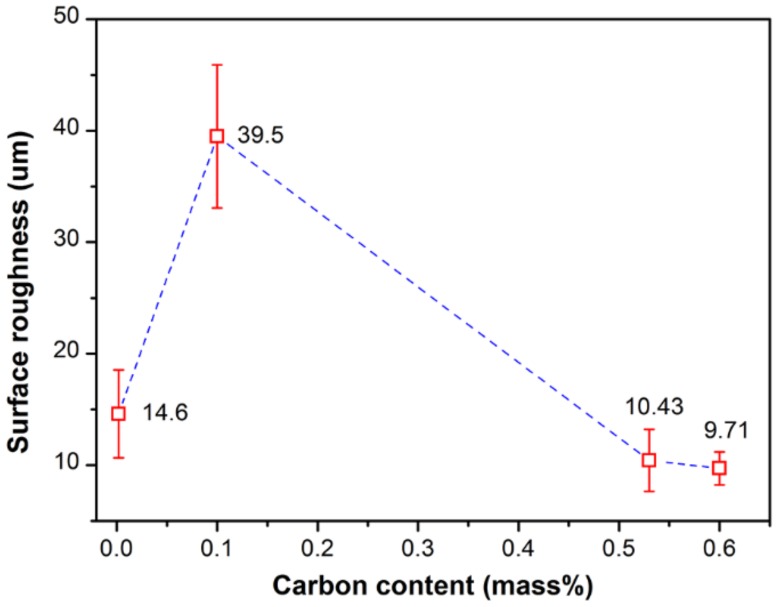
Relationship between the surface roughness and carbon content.

Figure 8 shows the relationship between the surface roughness (R_a_) and the carbon content. The maximum R_a_ value is found with the hypo-peritectic steel (R_a_ = 39.5 um), ~4 times greater than the ultra-low carbon steel and high carbon steels. The larger surface roughness indicates that it encounters severe uneven surface shrinkage deformation and may be vulnerable to surface defects. This result agrees with previous research regarding the effect of carbon content on the maximum reaction force and uneven shell growth of steels [32,39].

Here, measuring surface roughness using CSLM can only be carried out at room temperature. In order to elucidate changes in surface roughness, it is necessary to know the cause of surface deformation in steels. Generally, because the thermal contraction coefficient of a single-phase microstructure basically keeps a constant value in a certain range of temperature, uniform expansion or contraction does not give rise to surface morphology variation. Furthermore, different expansion or contraction coefficients of ferrite (α), pearlite (α+Fe_3_C), or cementite (Fe_3_C) after transformation lead to little surface shrinkage deformation for steels regardless of mixing ratio changes [40,41]. Therefore, only the phase transition will cause significant nonuniform volume contractions or expansions which result in surface roughness variation. This is why surface wrinkles can illustrate the phase transition occurring in the in-situ observational experiments. Nevertheless, the effect of phase transformation on surface roughness is different, and is related to temperature, material characters, transformation rate, and so on.

**Figure 9:**
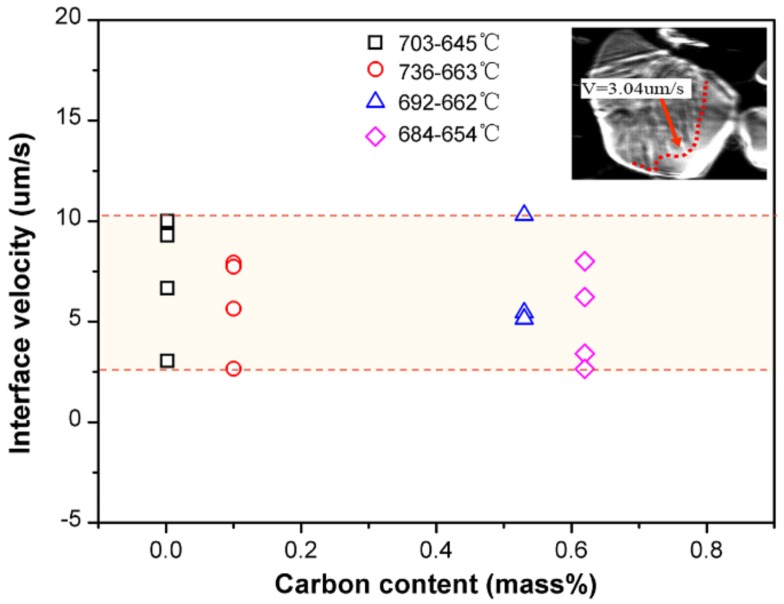
Phase transition interface velocity for steels at a low temperature range with a cooling rate of 1 °C/s.

For hypo-peritectic steel, the causes of surface roughness variation in this experiment are divided into three parts: δ–γ transformation, γ–α transformation, and the eutectoid transformation. Here, three factors are assumed to have a simple superposition effect. In order to evaluate the effect of the δ–γ transformation on the surface roughness, the following Equation (1) can be used:

(1) $$R_{a\left(\delta \to \gamma \right)}=R_{a}-\left(R_{a\left(\gamma \to \alpha \right)}+R_{a\left(\gamma \to \alpha +{\mathrm{Fe}}_{3}C\right)}\right)$$

where R*_a_* is the measured surface roughness, R*_a_*_(δ→γ)_, R*_a_*_(γ→α)_, R*_a_*_(γ→α+Fe_3_C)_ represent the surface roughness caused by δ–γ transformation, γ–α transformation, and eutectoid transformation, respectively. Accurate values of the latter two parts of the formula are difficult to obtain for hypo-peritectic steels, thus an indirect method is used. Figure 9 shows the phase transition rate for several steels at low temperature with the same cooling rate of 1 °C/s, where the rate is represented by the interface velocity by CSLM observation. The result shows that the reaction rate, ranging from 2.5 to 10 um/s, is significantly lower than the rate of massive transformation. The results indicate the stress changes are relatively similar for low temperature phase transitions. Thus, it is deduced that surface roughness produced by phase transition at low temperatures is similar for different steels if they have the same solidification sequence. To support this hypothesis, another high carbon steel with 0.65%C was . The surface roughness of the two high carbon steels were identical, 10.43 and 9.71 um, respectively (Figure 8).

**Figure 10:**
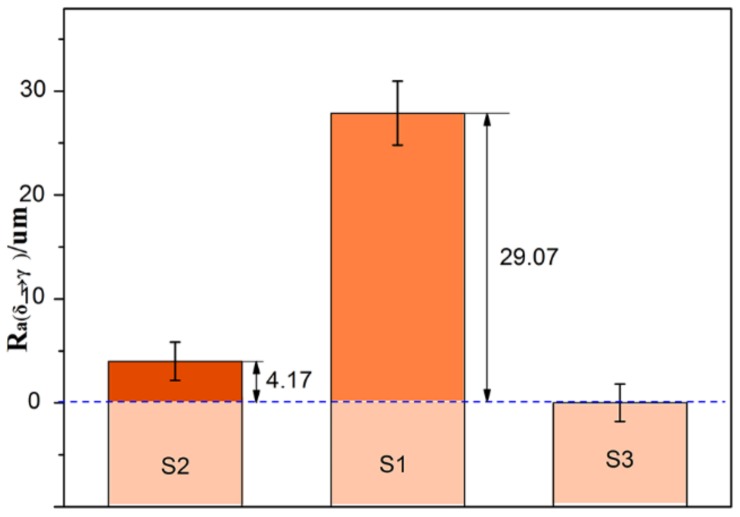
Surface roughness of δ–γ transformation, R*_a_*_(δ→γ)_.

Here, the R*_a_*_(δ→γ)_ for the hypo-peritectic steel, which is 29.07 um (Figure 10), can be calculated when the R*_a_* of S3 was selected as the standard surface roughness caused by phase transformation at low temperature. A sudden volume contraction on the surface resulting from massive transformation causes the deformation of the steel because of its lower strength during the peritectic transformation. In these conditions δ–γ transformation is the predominant cause of the surface roughness of hypo-peritectic steels. The R*_a_*_(δ→γ)_ for the high carbon steel is zero because there is no δ–γ transformation. For the ultra-low carbon steel, because the steel has fully solidified before the δ–γ transformation occurs, the higher tensile strength can resist shrinkage deformation. The R*_a_*_(δ→γ)_ of S2 is 4.17 um when neglecting the possible effect of a difference in transformation sequence at low temperature. Therefore, a rougher surface indicates that the thin shell was subjected to severe stress when the δ–γ transformation occurred, and that the solidifying shell in industrial practice may undergo serious deformation and then continue to cause uneven shell growth and thickness inhomogeneity of the solidification shell, resulting in cracks. According to this relationship, further investigation of the effect of phase transformation shrinkage on the integrity of thin shells under different conditions can be achieved via surface roughness variation.

## 4. Conclusions

CSLM equipment was used to study the variation of the surface roughness of steels subjected to large cooling rates at the initial solidification stage. The solidifying process of the sample surface was investigated and the surface roughness of the steels was measured and analyzed. The results show that the solidifying process in mold can be simulated using CSLM, and the solidifying processes are in accordance with the literature and the phase transition led to surface roughness variation. For the hypo-peritectic steel, δ–γ transformation greatly increases the surface roughness, which is much larger than that of other non-peritectic steels and it largely determines the surface deformation degree. Through calculating the R*_a_*_(δ→γ)_, the surface deformation due to δ–γ transformation is easily elevated for hypo-peritectic steels.

This article illustrates a new and effective method to further evaluate the effect of cooling on the δ–γ transformation contraction of hypo-peritectic steel, which can help us to further understand it under the non-equilibrium conditions. Further research could provide a reference for numerical work to select the boundary conditions and build a relevant model of the process. On the other hand, understanding the practical effect of the composition and cooling conditions on the phase transformation shrinkage will help us to further develop industrial strategies to improve the efficiency and quality of continuous casting production.

## Figures and Tables

**Table 1:** Chemical composition of steel samples.

Steel	Chemical Composition (mass %)									
	C	Si	Mn	S	P	Al	Ni	Ti	Cr	Fe
S1	0.102	0.211	1.22	0.001	0.011	0.039	0.120	0.017	0.179	bal.
S2	0.002	0.01	0.40	0.002	0.010	0.023	-	0.011	-	bal.
S3	0.54	0.151	0.51	0.010	0.015	0.021	0.101	0.014	0.151	bal.

**Table 2:** Comparison of temperature of nucleation and δ–γ transformation between theoretical and experimental tests.

Steel Samples/Temperature (°C)		S1	S2	S3
Equilibrium	δ nucleation	1518	1535	1486
	δ→γ transform	1487–1451	1393–1387	—
Actual (−20 °C/s)	δ nucleation	1462.5	1488.3	1338.5
	δ→γ transform	1328.5	1308.9–1303.1	—
